# Significant variations across European centres in implementing recommended guidelines for the paediatric gastroenterology endoscopy suite during the COVID-19 pandemic

**DOI:** 10.1097/PG9.0000000000000061

**Published:** 2021-05-27

**Authors:** Ilektra Athiana, Corinne Légeret, Patrick Bontems, Luigi Dall’Oglio, Paola De Angelis, Jorge Amil Dias, Bruno Hauser, Matjaž Homan, Priya Narula, Lorenzo Norsa, Salvatore Oliva, Alexandra Papadopoulou, Claudio Romano, Mike Thomson, Victor Vila-Miravet, Raoul I. Furlano

**Affiliations:** From the * University Children’s Hospital, Uppsala, Sweden; † University Children’s Hospital, Basel, Switzerland; ‡ Hopital Universitaire des Enfants Reine Fabiola, Brussels, Belgium; § Bambino Gesu Children’s Hospital, Rome, Italy; ∥ Centro Hospitalar Universitario S. Joao, Porto, Portugal; ¶ UZ Brussel KidZ Health Castle, Brussels, Belgium; # Department of Gastroenterology, Hepatology and Nutrition, University Children’s hospital, University Medical Center Ljubljana, Slovenia; ** Sheffield Children’s Hospital, Sheffield, United Kingdom; †† Pediatric Hepatology, Gastroenterology and Transplantation, ASST Papa Giovanni XXIII, Bergamo, Italy; ‡‡ Sapienza University of Rome, Rome, Italy; §§ Children’s Hospital Agia Sofia, Athens, Greece; ∥∥ Pediatric Gastroenterology and Cystic Fibrosis Unit Department of Human Pathology and Pediatrics University of Messina, Italy; ¶¶ Hospital Sant Joan de Déu, Barcelona, Spain

**Keywords:** children, COVID-19, endoscopy, ESPGHAN recommendations

## Abstract

The European Society for Paediatric Gastroenterology, Hepatology and Nutrition (ESPGHAN) published recommendations regarding protection for the paediatric endoscopist during the coronavirus 2019 (COVID-19) pandemic.The aim of this survey was to investigate whether European paediatric gastroenterology centres applied the recommendations and how this extraordinary situation was handled by the different centres.

**Results:**

Twelve paediatric European gastroenterology centres participated. Nine centres (75%) screened their patients for possible COVID-19 infection before the procedure, the same amount of hospitals changed their practice based on the ESPGHAN recommendations. Six-seven percentage of the centres reduced the staff in the endoscopy suite, 83% of the units used FFP2/3 masks and protective goggles during the procedure and 75% wore waterproof gowns.

**Conclusion:**

Uniform guidelines could not be applied by all European hospitals at a certain time point of the viral spread, as different regions of Europe were not only affected differently by COVID-19, but also had different access to personal protective equipment.

## Abstract 2

Supplemental Digital Content is available in the text.

**What Is Known?**

- Endoscopists are at risk to get infected by the coronavirus.
- ESPGHAN published recommendations regarding protection for paediatric endoscopists and endoscopy suite staff.

**What Is New?**

- This survey shows that 75% of participating centres changed their practice based on the ESPGHAN guidelines.
- Uniform guidelines may be challenging to implement as countries were differently affected by COVID-19 and had different access to protective equipment.

A novel coronavirus (SARS-CoV-2), first identified in early December 2019 in Wuhan, China, was found to cause the coronavirus 2019 (COVID-19) pandemic, affecting over 200 countries and territories. SARS-CoV-2 is a positive-sense, single-stranded RNA virus (1). To reduce the transmission rate, authorities instructed among other directives to reduce social gatherings to a minimum and maintain distances in between people. As endoscopists are at particular risk given the recently identified exposure of the endoscopist’s face to biological material during the procedure (2), the North American Society for Pediatric Gastroenterology, Hepatology and Nutrition and the European Society for Paediatric Gastroenterology, Hepatology and Nutrition (ESPGHAN) published recommendations early in the pandemic (3,4) for paediatric endoscopists and theatre staff regarding personal protection for the paediatric endoscopist and theatre staff before and during endoscopic procedures: most importantly, it was recommended to put all elective procedures on hold, as upper gastrointestinal endoscopies are an “aerosol-generating procedure” whilst during ileocolonoscopies, COVID-19 can be excreted in stools. Urgent diagnostic and interventional endoscopies should not be deferred, but theatre staff was recommended to wear a full personal exposure protection package including an FFP3 mask or equivalent and accessories used should immediately be disposed. A risk to benefit balance should occur if the patient is known to be carrying SARS-CoV-2 or to have had recent contact.

Different European countries not only have a different incidence of affected people but also experience different barriers against the adoption of these recommendations (e.g., shortage of medical resources). Our aim was to investigate whether paediatric gastroenterology centres throughout Europe did implement these recommendations and how the current situation was handled by the different centres.

## METHODS

This study was conducted as a survey between April 22, 2020 and May 7, 2020.

Members of the ESPGHAN Endoscopy special interest group (5) and other paediatric gastroenterology centres were contacted by email and were invited to participate in this survey. Participants were informed that survey completion was voluntary, completion of a survey implies consent and results would be reported anonymously and in aggregate.

A questionnaire, containing questions regarding characteristics of the endoscopy unit and 12 questions related to the current situation caused by the COVID-19 pandemic (See Supplemental Digital Content Table 1, http://links.lww.com/PG9/A26), including COVID-19 screening processes and use of personal protective equipment (PPE), was sent out. The questionnaire was designed in a responder-friendly manner with clear language and the possibility to select the answers to ensure a complete compilation of the questionnaire.

Data were transferred into an Excel table, analysed using descriptive statistics.

The present study was approved by the local ethical committee (Ethics committee of Northwest Switzerland, EKNZ, trial number 2020-01209). Furthermore, the study was conducted in accordance with the ethical principles laid down in the Declaration of Helsinki and its later amendments.

## RESULTS

### Characteristics of Endoscopy Units

Twelve paediatric gastroenterology centres throughout Europe (Belgium, Greece, Italy, Portugal, Slovenia, Spain, Switzerland, and United Kingdom) were invited and all participated. Five participating units (5/12, 41.7%) have a volume of 500-1000 endoscopies/year, followed by four units (4/12, 33.3%) performing 100-500 endoscopies/year and three units (3/12. 25%) with a performance of >1000 endoscopies/year. Paediatric gastroenterologists perform endoscopies in all centres (see Table 1). In eight (8/12, 66.6%) of the centres, endoscopies are additionally performed by paediatric gastroenterology trainees. In four (4/12, 33.3%) of the units, endoscopies are performed by paediatric surgeons and in three of these centres paediatric surgery trainees are performing endoscopies as well. In only one centre (1/12, 8.3%), additionally adult gastroenterologists and their trainees perform endoscopies.

### Screening of Patients and Staff

All (12/12) centres had to cancel or postpone elective endoscopies due to COVID-19 outbreak, mainly beginning in mid-March 2020. Nine centres (9/12, 75%) screened their patients for possible COVID-19 infection before the endoscopic procedures. No unit tested the endoscopy staff routinely, but all did in case the staff presented with typical symptoms (fever, cough, muscle ache, shortness of breath, sore throat, sudden loss of smell and/or taste, gastrointestinal symptoms). Endoscopy suite staff were not involved in any endoscopic procedures while the test results were still pending.

### Infection Prevention in Endoscopy Units due to COVID-19

In more than half (7/12, 58.3%) of the paediatric endoscopy units 3-5 healthcare professionals are present during an endoscopy, in four centres (4/12, 33.3%) between 5 and 10 staff members are involved and in one centre (1/12, 8.3%) less than three people are present.

Eight centres (8/12, 66.6%) reduced the staff in the endoscopy suite due to the pandemic, see Table 1.

**TABLE 1.** Overview of the 12 participating centres

Centre	Average endoscopies/year			Patients screened prior to endoscopy	Staff involved changed during pandemic	Changes based on ESPGHAN recommen-dations	Local guidelines	Personal protective equipment used during endoscopy							
	100-500	500-1000	>1000					Protective goggles	Face shield	Single pair of gloves	Double pair of gloves	Surgical mask	FFP2/3 or N95 mask	Waterproof gown	other
1			X	No	Yes	Yes	Yes	X	X	X			X	X	
2			X	Yes	No	No	Yes	X	X	X		X	X		
3	X			Yes	Yes	No	No		X		X		X	X	
4	X			No	Yes	No	Yes	X	X		X		X		
5	X			No	Yes	Yes	Yes	X		X			X	X	
6		X		Yes	Yes	Yes	No	X			X		X	X	Shoe covers
7		X		Yes	No	Yes	Yes	X	X			X	X	X	
8	X			Yes	No	Yes	Yes	X		X			X	X	
9		X		Yes	No	Yes	Yes	X	X	X		X	X	X	
10			X	Yes	Yes	Yes	No	X			X		X	X	
11		X		Yes	Yes	Yes	Yes	X	X		X		X	X	
12		X		Yes	Yes	Yes	No		X	X		X	X	X	

ESPGHAN = European Society for Paediatric Gastroenterology, Hepatology and Nutrition.

Nine centres (9/12, 75%) changed their practice based on the ESPGHAN COVID-19 endoscopy statement (5). Eight centres (8/12, 66.6%) had guidelines provided by their local institution on which cases are considered emergent/urgent and/or can be performed.

Five endoscopy units (5/12, 41.6%) added additional disinfecting steps: total disinfection of the suite in between patients and disinfection with virucidal agents in high-risk patients, these practices were mainly followed by centres with high infection rates.

Regarding the use of PPE, nine paediatric endoscopy centres (9/12, 75%) were using protective goggles during endoscopic procedures. All centres (12/12, 100%) were using FFP2/3 or N95 masks. Eight centres (8/12, 66.7%) were using face shields and 10 centres (10/12, 83.3%) provided endoscopists with waterproof gowns. From the above-mentioned centres, two reported the use of PPE only during endoscopies performed on patients who had tested positive for COVID-19 and one centre reported the use of FFP2/3 masks only for highly suspected or COVID-19 positive patients.

## DISCUSSION

Our survey reveals the burden for paediatric gastroenterology departments caused by the outbreak of the COVID-19 pandemic. All centres had to cancel elective procedures and limit endoscopies to urgent cases only. Although ESPGHAN reacted quickly to this exceptionally difficult situation and provided clinicians with early recommendations during the evolution of the viral spread, not all centres followed those guidelines, and most centres complied in addition with the recommendations of their own institutions and/or governmental directives.

All centres strictly adhered to the ESPGHAN guidelines regarding cancelling elective endoscopies, whilst the usage of goggles/face shields and FFP 2/3 masks was handled differently. This might be explained by the fact, that the survey was taken at the beginning of the pandemic, where many countries had different access to PPE supplies. Furthermore, different countries were affected differently by the pandemic. The last point is underlined by the result of this survey, that centres of strongly affected countries implemented extra disinfecting steps (cleaning the suite, etc.), which were not specifically recommended by ESPGHAN.

Uniform guidelines were not applied by all European hospitals at a certain timepoint of the COVID-19 pandemic as different regions of Europe were not only affected differently, at different timepoints but also had different access to PPE and needed to follow local/national recommendations. Efforts should be undertaken by the respective guideline-producing societies for assisting the implementation of the societal guidelines by direct communication with hospitals and local and/or national authorities/societies also taking into consideration local differences.

It is a fact that it is extremely difficult to define the right timepoint to publish guidelines or statements during a pandemic. The reasons for these difficulties are the differing impact of the virus in each country or region, the different rules given by the governments and the compliance to those rules and that these may change rapidly during a pandemic.

Centres from regions, which had high numbers of infected people, tended to screen their patients for COVID-19 before performing the endoscopies, whilst centres from countries, which were only mildly affected, abstained from it. This reflects different approaches of countries, who have been confounded differently by COVID-19 at the point of the survey.

Consistently, most centres reduced the amount of staff present during endoscopic procedures and provided staff with PPE as recommended by ESPGHAN. Wearing single or double pair of gloves was handled very differently, as well as disinfecting the endoscopy unit in between the procedures.

A clear limitation of this survey is that only selected European centres participated and it was already conducted in April 2020, not only shortly after publication of the ESPGHAN recommendations but at a point where not all European countries have already really been affected by the virus. Another disadvantage is, that it is only a snapshot (implementation of the guidelines) of a troublesome, constantly changing situation, which affected and still affects different countries in different ways (different amount of infected patients, delivery issues) at different timepoints, therefore results of this survey can only be published in a descriptive way.

## CONCLUSION

The global situation caused by COVID-19 changed so rapidly, that hospitals had to react immediately to protect staff and patients and could not wait for guidelines to be published. The burden of COVID-19 on endoscopy units is substantial—not only does the paediatric gastroenterologist need to make the decision about which endoscopies are urgent, but also how to protect the staff with the medical supply available for PPE. In this study major variations regarding the implementations of the ESPGHAN guidelines were found, mainly depending on how hard and at which timepoint the country was affected by COVID-19.

Time and upcoming studies will also show if delayed elective endoscopic procedures will have an impact on children’s health and/or on the health economy.

**FIGURE 1.**
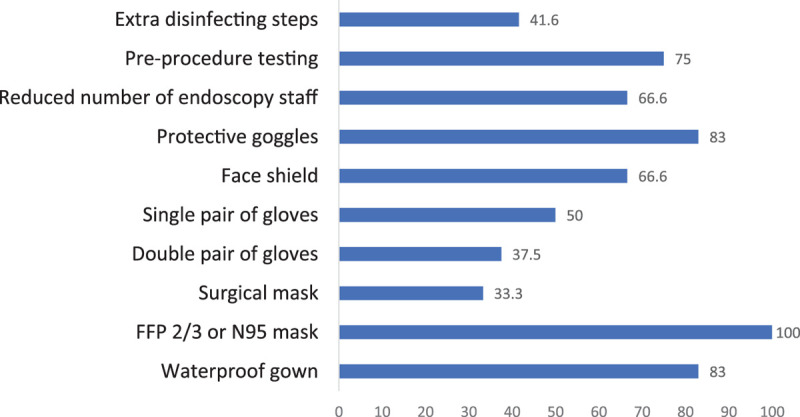
Use of PPE in the 12 endoscopy centres (presented in percentage). PPE = personal protective equipment.

## Supplementary Material


