# Supplementary material for: Significant variations across European centres in implementing recommended guidelines for the paediatric gastroenterology endoscopy suite during the COVID-19 pandemic
Source: JPGN Rep. 2021 May 27;2(3):e061. doi: 10.1097/PG9.0000000000000061 (PMC8162040; doi:10.1097/PG9.0000000000000061)
Supplement: Supplementary file 1 [file pg9-2-e061-s001.pdf]

Supplementary table – List of questions in the questionnaire

1. Participating center:

2. City:

3. Country:

4. Local principal investigator:

5. Contact details:

6. Doctors performing endoscopy at your center:
   - ☐ Paediatric gastroenterologist
   - ☐ Paediatric gastroenterology trainee
   - ☐ Paediatric surgeon
   - ☐ Paediatric surgeon trainee
   - ☐ Adult gastroenterologist
   - ☐ Adult gastroenterology trainee
   - ☐ Other (please specify)

7. Average number of endoscopies per year at your center?
   - ☐ <100
   - ☐ 100-500
   - ☐ 500-1000
   - ☐ >1000

8. Did your center postpone/cancel elective endoscopies due to COVID-19 outbreak?
   - ☐ Yes
   - ☐ No

   If yes, from which date?

9. When did the quarantine start in your city?

10. Were the patients tested for COVID-19 before the endoscopic procedures?
    - ☐ Yes
    - ☐ No

    If yes, from which date?

11. Were the endoscopy staff routinely tested for COVID-19?
    - ☐ Yes
    - ☐ No

    If yes, from which date?

12. Were the endoscopy staff tested for COVID-19 if they have shown symptoms?
    - ☐ Yes
    - ☐ No

    If yes, from which date?

13. Did the number of staff involved in the endoscopy suite change during the pandemic?
    - ☐ Yes
    - ☐ No

14. Staff involved at the endoscopy suite:
    - ☐ Endoscopist
    - ☐ Anesthesiologist
    - ☐ Nurse anesthetist
    - ☐ Endoscopy nurse
    - ☐ Other

15. What is the average number of staff involved during an endoscopy at your center?
    - ☐ 0-3
    - ☐ 3-5
    - ☐ 5-10

16. Did you make any changes in your personal practice based on the ESPGHAN COVID-19 endoscopy statement?
    - ☐ Yes
    - ☐ No

17. Has your institution set out guidelines as to which cases are considered emergent/urgent and/or can proceed?
    - ☐ Yes
    - ☐ No

18. Do you have any additional disinfecting steps of the endoscopy suite due to COVID-19?
    - ☐ Yes
    - ☐ No

    If yes, please specify

19. Specify the personal protective equipment (PPE) used during endoscopic procedures:

- ☐ Protective goggles
- ☐ Face shield
- ☐ Single pair of gloves
- ☐ Double pair of gloves
- ☐ Surgical mask
- ☐ FFP2/3 mask
- ☐ Waterproof gown
- ☐ Other (please specify which)
